# Suitability of PSA-detected localised prostate cancers for focal therapy: experience from the ProtecT study

**DOI:** 10.1038/bjc.2011.314

**Published:** 2011-08-23

**Authors:** J W F Catto, M C Robinson, P C Albertsen, J R Goepel, M F Abbod, D A Linkens, M Davis, D J Rosario, A Y Warren, M Varma, D F Griffiths, K M Grigor, N J Mayer, J D Oxley, N S Deshmukh, J A Lane, C Metcalfe, J L Donovan, D E Neal, F C Hamdy

**Affiliations:** 1 Academic Urology Unit and Institute for Cancer Studies, University of Sheffield, Sheffield, UK; 2 Department of Cellular Pathology, Royal Victoria Infirmary, Newcastle, UK; 3 Department of Surgery, University of Connecticut Health Center, Hartford, CT 06030, USA; 4 Department of Pathology, Royal Hallamshire Hospital, Sheffield, UK; 5 School of Engineering and Design, Brunel University, Uxbridge, UK; 6 Department of Automatic Control and Systems Engineering, University of Sheffield, Sheffield, UK; 7 School of Social & Community Medicine, University of Bristol, Bristol, UK; 8 Department of Pathology, University of Cambridge, Cambridge, UK; 9 Department of Pathology, University Hospital of Wales, Cardiff, UK; 10 Department of Pathology, Western General Hospital, Edinburgh, UK; 11 Department of Pathology, University of Leicester, Leicester, UK; 12 Department of Cellular Pathology, Southmead Hospital, Bristol, UK; 13 CRUK Institute of Cancer Studies, University of Birmingham, Birmingham, UK; 14 Department of Oncology, University of Cambridge, Cambridge, UK; 15 Nuffield Department of Surgical Sciences, University of Oxford, Headley Way, Headington, Oxford OX3 9DU, UK

**Keywords:** prostate cancer, unifocal, regional ablation, prostatectomy

## Abstract

**Background:**

Contemporary screening for prostate cancer frequently identifies small volume, low-grade lesions. Some clinicians have advocated focal prostatic ablation as an alternative to more aggressive interventions to manage these lesions. To identify which patients might benefit from focal ablative techniques, we analysed the surgical specimens of a large sample of population-detected men undergoing radical prostatectomy as part of a randomised clinical trial.

**Methods:**

Surgical specimens from 525 men who underwent prostatectomy within the ProtecT study were analysed to determine tumour volume, location and grade. These findings were compared with information available in the biopsy specimen to examine whether focal therapy could be provided appropriately.

**Results:**

Solitary cancers were found in prostatectomy specimens from 19% (100 out of 525) of men. In addition, 73 out of 425 (17%) men had multiple cancers with a solitary significant tumour focus. Thus, 173 out of 525 (33%) men had tumours potentially suitable for focal therapy. The majority of these were small, well-differentiated lesions that appeared to be pathologically insignificant (38–66%). Criteria used to select patients for focal prostatic ablation underestimated the cancer's significance in 26% (34 out of 130) of men and resulted in overtreatment in more than half. Only 18% (24 out of 130) of men presumed eligible for focal therapy, actually had significant solitary lesions.

**Conclusion:**

Focal therapy appears inappropriate for the majority of men presenting with prostate-specific antigen-detected localised prostate cancer. Unifocal prostate cancers suitable for focal ablation are difficult to identify pre-operatively using biopsy alone. Most lesions meeting criteria for focal ablation were either more aggressive than expected or posed little threat of progression.

As a consequence of repeated testing for prostate-specific antigen (PSA), prostate cancer is now the most common male malignancy in North America and Europe (Jemal *et al*, 2010). Of the 217 730 men diagnosed with this disease in the United States, approximately half were classified as low-risk disease (Stamey *et al*, 2004; Cooperberg *et al*, 2007). Recent data from the European Randomized Screening for Prostate Cancer Trial suggest that many of these cases pose little threat of disease progression and that only 1 of every 37–48 men diagnosed by PSA testing may benefit from aggressive treatment (Roobol *et al*, 2009). Although this number-needed-to-treat may reduce as the length of follow-up increases within this trial, it is likely that overtreatment will still be prevalent for screen-detected cancers.

The concern surrounding the potential for overtreatment of localised prostate cancer has led many clinicians to propose alternative treatment strategies. Some clinicians have advocated active surveillance, but many patients are hesitant to accept this approach because of the fear of disease progression (Denberg *et al*, 2006). Focal prostate ablation has recently emerged as a less invasive alternative to radical prostatectomy and a more acceptable approach than active surveillance (Sartor *et al*, 2008).

The objective of focal ablative intervention is to destroy cancerous prostate tissue without targeting the entire gland. High-intensity frequency ultrasound (HIFU), photothermal and cryotherapy are promising techniques that offer the potential of lower morbidity when compared with radical surgery, external beam radiation or brachytherapy. Unfortunately few centres have reported long-term outcomes to validate this approach and there are no reported controlled trials that confirm the efficacy of these treatments (Onik *et al*, 2008; Challacombe *et al*, 2009; Lindner *et al*, 2009; Warmuth *et al*, 2010).

One major concern surrounding the efficacy of focal therapy stems from the observation that many prostate cancers are not solitary. Depending upon the patient population selected, how the tissue was processed and which definitions are used, as many as 50–87% of prostate cancers have multiple sites within the gland (Meiers *et al*, 2007). Many of these lesions cannot be detected pre-operatively by biopsy or imaging.

The present study is based on a cohort of men with screen-detected prostate cancer, who then underwent radical prostatectomy as part of a randomised controlled trial (Donovan *et al*, 2003). Our aims were, first, to estimate the proportion of men with screen-detected prostate cancer for whom focal therapy would be appropriate. Second, to establish whether measures taken during the diagnostic process could be used to identify those men for whom focal therapy would be a suitable treatment of their prostate cancer. Third, to detail the pathological characteristics of tumours suitable for focal ablation.

## Patients and methods

### Patient population

Data used in this analysis were prospectively collected from 525 men who underwent radical prostatectomy within the ProtecT trial (NCT00632983; HTA 96/20/06; HTA 96/20/99) (inclusion criteria described in: Donovan *et al*, 2003; Lane *et al*, 2010), a large randomised trial testing the efficacy of prostate cancer treatment in men detected through population-based PSA testing. Men recruited into ProtecT study were identified from community medical practices and aged 50–69 years at initial invitation with no prostatic symptoms. Men included in this study were those randomised to or choosing surgery after having been diagnosed with prostate cancer following a 10 core trans-rectal biopsy performed because of a single-serum PSA value between 3 and 19.9 ng ml^−1^, in whom complete pathological records were available at the time of this analysis. Men included in the ProtecT trial were used, as opposed to those from a tertiary center case series, to avoid biasing the sample towards high-risk tumours. The study population is described in Table 1.

### Pathological analysis

Pathologic assessment of needle biopsy prostate cores and radical prostatectomy specimens was made by specialist urological pathologists using standardized protocols and agreed reporting pro-formas. Prostate biopsy specimens were classified as predicting insignificant disease if they met Epstein's criteria (PSA density <0.15 ng ml^−1^, Gleason score ⩽6, two cores or less with cancer and no core containing more than 50% cancer) (Epstein *et al*, 1994) and predicting low risk if they met the following criteria; PSA ⩽20 ng ml^−1^, Gleason score ⩽6, ⩽50% positive cores, ⩽20 mm cancer and at least 40 mm benign tissue (Kattan *et al*, 2003; Steyerberg *et al*, 2007). For each tumour, we calculated the pre-operative risk of indolent disease using the Kattan–Steyerberg Nomogram (Steyerberg *et al*, 2007) and identified tumours that satisfied the proposed consensus criteria predicting tumour unifocality (i.e., PSA ⩽10 ng ml^−1^ or PSAD⩽0.15 ng ml^−1^ g^−1^, Gleason sum score ⩽6, cumulative cancer volume ⩽10 mm, ⩽7 mm of cancer within one core and ⩽33% of needle cores with cancer) (Sartor *et al*, 2008).

Radical prostatectomy specimens were completely embedded, mapped and the tumour apportioned between five regions: anterior to the urethra, posterior left, posterior right, posterior apical and posterior base. Tumours located entirely or mostly (no more than 20% of volume in an adjacent region) within one prostate region were defined as solitary tumours. Tumour volume was calculated using a three-dimensional method (Chen *et al*, 2003). Multiple tumours were identified as those with discrete malignancies separated by one low-power field diameter. The tumour with the most advanced stage, highest grade or highest volume was defined as the dominant tumour. Insignificant tumours in the prostatectomy specimens were classified according to the Epstein's criteria (⩽0.5 ml tumour volume, Gleason score ⩽6, organ confined) (Epstein *et al*, 2005) and more liberal criteria (any volume, pT2, Gleason score ⩽6, organ confined, no adverse features (e.g., vascular invasion)) (Sengupta *et al*, 2008; Lee *et al*, 2009). Solitary single cancers and multifocal tumours with a dominant solitary focus and insignificant secondary foci were defined as those suitable for focal therapy.

### Statistical analysis

Measures made before surgery were evaluated for their ability to identify those suitable for focal therapy using univariable logistic regression, a multivariable model determined by backward selection and Harrell's concordance index (Harrell *et al*, 1982; Catto *et al*, 2009). For regression, we analysed age of the patient, serum PSA, prostate volume, PSA density, Gleason grade, digital rectal examination findings and measures of tumour extent in the biopsy core. Independence between variables was determined before inclusion (*r*<0.5) and separate models used for collinear variables. The predictive power of the final multivariable models was evaluated using sensitivity, specificity, positive predictive value, and the area under the receiver operating characteristic curve. All tests were two-tailed, performed within SPSS statistical software (Version 18.0, 2010, SPSS Inc., Chicago, IL, USA) and significance was defined as a *P*-value <0.05.

## Results

**Figure 1:**
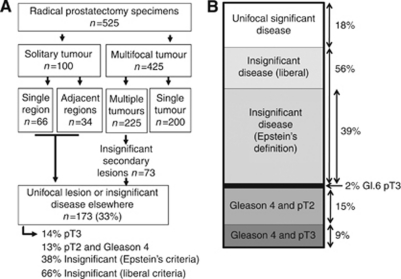
Tumour focality in prostate cancer. (**A**) Flowchart of tumour focality within the samples described within this report. (**B**) The pathological details of tumours fulfilling the criteria for focal therapy. The thick black line represents the 2% of tumours that are pT3 and Gleason 3+3=6.

We report data from 525 men within the ProtecT trial who underwent radical prostatectomy with curative intent. At diagnosis, the average patient age was 61 years (s.d. 5), the average serum PSA was 6.1 ng ml^−1^ (s.d. 3.6) and the average prostate size was 34.4 ml (TRUS volume, s.d. 15.2). Of the 525 men, 512 (97.5%) harboured Gleason score 6 or 7 disease, 496 (94.5%) had 10 or more biopsy cores assessed and 423 (80.6%) were diagnosed following the first biopsy. Solitary prostate cancers were identified in the prostatectomy specimens of 100 (19%) men (Table 1, Figure 1A). Of these, tumour was located in a single region in 66 and in two adjacent regions in 34 men. The remaining 425 men had multifocal lesions consisting of either a diffuse dominant tumour (200 men, 47%) or multiple cancers (225 men, 53%). In these men, the secondary lesions were insignificant in 33% (73 out of 225) and hence may be suitable for focal treatment (Eggener *et al*, 2007). Thus, 33% of men (173 out of 525) had tumours suitable for focal ablation (i.e., unifocal significant tumours).

Men with unifocal significant tumours had a lower Gleason score (*χ*^2^-test, *P*<0.001), lower stage (*P*<0.001), fewer cores positive at biopsy (mean=11% of cores had cancer (95% CI: 6–16%)) *vs* 33% for multifocal tumours (28–38%), ANOVA *P*<0.001), less disease within these cores (mean cumulative length of unifocal cancer=6 mm (95% CI: 3–10 mm) *vs* 20 mm for multifocal disease (16–25 mm, ANOVA *P*<0.001)) and less vascular invasion in the prostatectomy specimen than those with multifocal disease (*χ*^2^-test, *P*=0.06) (Table 1). Of note, multifocal tumours were found more easily than unifocal ones (84% detected at first biopsy *vs* 73% for unifocal cancers, *χ*^2^-test, *P*=0.007). Multivariable analysis revealed that tumour unifocality was most associated with pathologically defined insignificant disease and tumour volume (*P*<0.0001). Focal ablation is not recommended for tumours with poorly differentiated components or with capsular invasion (Sartor *et al*, 2008). In total, 118 out of 173 (68%) of unifocal cancers were Gleason score ⩽6 and pT2. Of these, 69 out of 118 (58%) and 118 out of 118 (100%) were pathologically insignificant according to Epstein's and the liberal definitions, respectively. Therefore, between 56 out of 123 (47%) and 9 out of 123 (7%) of unifocal tumours were Gleason 6, pT2 and pathologically significant, depending upon the definition of pathological insignificance.

Several pre-operative measures were associated with the presence of a unifocal cancer (Table 2). Owing to covariate correlation (*r*>0.5), biopsy features describing tumour extent (bilateral disease, total and maximal tumour length and percentage of cores with cancer) and insignificant cancer definitions were incorporated separately into models. The likelihood of unifocal disease increased with a high nomogram score (HR 4.3 (95% CI: 1.8–10.5)), in tumours fulfilling the focal treatment criteria (HR 2.4 (1.5–3.8)) and those appearing as low risk on biopsy (HR 2.9 (1.8–4.4)). In contrast, tumour bilaterality on biopsy (HR 0.27 (0.17–0.42)), increasing numbers of involved cores (HR 0.49 (0.04–0.9)) and high Gleason grade (HR 0.6 (0.4–0.9)) reduced the risk of unifocal disease. When compared with other parameters, bilateral disease on biopsy was the feature most strongly associated with multifocal disease.

On the basis of this information available following prostate biopsy, 130 (25%) of the 525 men were predicted as being suitable for focal ablation (Table 3, Figure 1B). A review of their surgical specimens showed that 92 (71%) of these men harboured well-differentiated tumours. In total, 39% met Epstein's criteria and 56% met the more liberal criteria for insignificant disease. Overall, 31 (24%) had Gleason grade 4 elements and 14 (11%) had extracapsular extension. Multifocal disease was present in 106 out of 130 (82%) of these men. Of these, the second focus was pathologically insignificant in 61 out of 106 (57%). If these criteria were used to direct focal ablation within our patients, they would have correctly identified 18% of men with clinically significant solitary tumours; but would have also included 56% of men with pathologically insignificant tumours and 26% of men with poorly differentiated or locally advanced cancers (Figure 1A).

The criteria used to select men for focal prostate ablation did not reliably identify solitary lesions (Concordance index (C-index)=0.50), pathologically significant disease (C-index=0.64–0.71), tumour stage (C-index=0.65), tumour grade (C-index=0.68) or tumour volume (C-index=0.75). Men meeting these criteria were likely to harbour smaller tumours in larger prostates when compared with the other men in the study cohort (Table 3). In fact, the criteria developed to identify men for focal treatment were more likely to select patients on the basis of prostate volume (C-index=0.76) rather than tumour parameters.

## Discussion

Our study demonstrates that focal prostate ablation is an inappropriate therapy for the majority of men presenting with PSA-detected localised prostate cancer. We found that measures available following prostate biopsy could not reliably identify men with solitary tumours amenable for focal therapy. Among men who do harbour well-differentiated solitary lesions, the majority are small lesions that are probably best managed by active surveillance.

Consensus criteria developed by experts to identify men for focal prostate ablation mostly relied on prostate weight and tumour volume and thus did not reliably identify clinically significant solitary prostate lesions. Of greater concern, these criteria included as many as one quarter of the men harbouring Gleason grade 4 disease or with capsular invasion (Figure 1B).

When we evaluated all the measures available following prostate biopsy, we found that markers of tumour extent could accurately identify men who should not receive focal prostate ablation, but we were unable to identify reliably those men with solitary lesions who would benefit from focal therapy. For example, when more than 50% of biopsy cores were involved with tumour, we found that most of these tumours (>95%) were multifocal. In contrast, multifocal disease was present in 75% of men with only 1 mm of cancer in one biopsy core. Our data support the concept of template-mapping protocols before performing regional ablation, and suggest the need for formal prospective evaluation of this method.

Our study may be limited in that it underestimates the proportion of solitary or insignificant cancers present in contemporary European or North-American practices. Reports describing these populations, however, reveal a similar proportion of solitary, multifocal or bilateral lesions to that we observed in our data (Table 4). Frequent testing for PSA may find prostate cancers earlier in their natural history, but the anatomical distribution of these cancers appears to be comparable to those identified in the United Kingdom.

In summary, our analysis suggests that the majority of men presenting with prostate cancer following testing for PSA are inappropriate candidates for focal prostate ablation. Men who harbour solitary prostate cancers most often have insignificant disease that might be best managed by active surveillance (van den Bergh *et al*, 2009), whereas men harbouring multi-focal prostate cancers usually have clinically significant disease that requires more extensive treatment.

## Figures and Tables

**Table 1:** Radical prostatectomy features of unifocal and multifocal tumours

	**Unifocal significant tumours**		**Multifocal tumours**			
	***n***	**%**	***n***	**%**	***P*-value^a^**	**Multivariable *P*-value^b^**
Total	173		352			
Age (years, mean (s.d.))	61.4 (5.0)		60.7 (4.9)		0.9	
						
*Differentiation*						
Gleason 5 or 6	132	78.6	121	35.2		
Gleason 3+4=7	28	16.7	174	50.6		
Gleason 4+3=7	4	2.4	38	11.0		
Gleason 8–10	4	2.4	11	3.2		
Missing	5	2.8	8	2.3	<0.001	0.3
						
*Stage*						
pT2	149	86.4	212	60.3		
pT3	24	13.6	140	39.7	<0.001	0.2
						
*Insignificant cancer* c						
Insignificant (Epstein)	69	39.9	2	0.6	<0.001	<0.001
Insignificant (Liberal)	118	68.2	39	11.1	<0.001	<0.001
						
*Other pathological features*						
Vascular invasion	0	0.0	15	4.3	0.001	0.1
HGPIN	150	87.7	331	94.3	0.09	0.3
Seminal Vesicle invasion	1	0.6	29	8.3	<0.001	0.3
						
*Tumour volume (ml, mean (s.d.))*						
Total	1.18 (1.7)		4.33 (9.7)		<0.001	<0.001
Dominant	1.05 (1.7)		3.64 (7.8)		<0.001	0.001
Secondary	0.16 (0.4)		0.77 (3.7)		0.08	0.4
Tertiary	0.05 (0.1)		0.15 (0.3)		0.02	0.8
						
*Nomogram score (mean (s.d.))* d						
Risk of indolent cancer	0.75 (0.2)		0.58 (0.3)		<0.001	0.9

Abbreviations: HGPIN=high-grade prostatic intraepithelial neoplasia; s.d.=standard deviation.

a *χ*^2^-test for all variables except tumour volume, and age (*t*-test).

b Backward elimination logistic regression using parameters with univariable significance.

c Histological definition from the prostatectomy specimen: Epstein's: < 0.5 ml tumour volume, Gleason score < 6, organ confined; liberal: pT2, Gleason score <6, organ confined, no adverse features.

d Score calculated using Kattan–Steyerberg nomogram (Steyerberg *et al*, 2007).

**Table 2:** Pre-operative features of unifocal and multifocal prostate cancers

	**Unifocal**		**Multifocal**					**HR 95% CI**	
	**Mean**	**s.d.**	**Mean**	**s.d.**	***P*-value Univariable analysis^a^**	***P*-value Multivariable analysis^b^**	**HR**	**Low**	**High**
*Interval data*									
Serum PSA at diagnosis (ng ml^−1^)	5.5	3.1	6.4	3.8	0.005	0.07			
TRUS prostate volume (ml)	36.8	16.2	33.2	14.5	0.014	0.3			
PSA density	0.17	0.14	0.22	0.16	0.001	0.08			
Total length of tissue (mm)	131.3	35.7	130.7	33.9	0.9				
Positive cores (% of total)	11	31.4	33	47.2	<0.001	0.001	0.49	0.04	0.9
Cumulative cancer length (mm)	6	24.5	20	40.4	<0.001	0.09			
Maximal tumour length (mm)	3.1	2.7	5.1	5.7	<0.001	0.08			
Nomogram prediction	0.75	0.24	0.58	0.29	<0.001	<0.001	4.3	1.8	10.5
									
*Categorical data*	n	*%*	n	*%*					
Gleason score									
Gleason 5–6	151	87.3	239	67.9					
Gleason 7	20	11.5	102	29.2					
Gleason 8–10	2	1.2	10	2.9	<0.001	0.009	0.6	0.4	0.9
									
Number of biopsy sessions									
One	126	72.8	297	84.4					
Two or more	47	27.2	55	15.6	0.007	0.1			
									
Number of biopsy cores									
10 or fewer	131	75.7	297	84.4					
More than 10	42	24.3	55	15.6	0.3				
									
Lobes involved 2008 Focal therapy criteria, insignificant cancer									
Bilateral	36	20.8	164	46.6	<0.001	<0.001	0.27	0.17	0.42
Yes	68	42.8	62	18.2	<0.001	0.002	2.4	1.5	3.8
Epstein's criteria	59	37.1	51	15.0	<0.001	0.9	0.9	0.83	0.97
Low risk	134	77.5	171	48.6	<0.001	0.001	2.9	1.8	4.4

Abbreviations: CI=confidence interval; HR=Hazards ratio; PSA=prostate-specific antigen; TRUS=transrectal ultrasound.

a ANOVA for continuous and *χ*^2^-test for categorical data.

b Backward elimination logistic regression.

**Table 3:** Pathological features of tumours fulfilling pre-operative criteria for focal ablation

	**Suitable for focal ablation**		**Unsuitable for focal ablation**		
	***n***	**%**	***n***	**%**	***χ*^2^-test, *P*-value**
Total	130		370		
Age (mean (s.d.))	61.3 (5.1)		60.6 (4.9)		
					
*Differentiation*					
Gleason 5 or 6	92	70.8	143	38.6	
Gleason 3+4=7	30	23.1	166	44.9	
Gleason 4+3=7	1	0.8	41	11.1	
Gleason 8–10	0	0.0	14	3.8	
Missing	7	5.4	6	1.6	<0.001
					
*Stage*					
pT2	114	87.7	216	58.4	
pT3	14	10.8	145	39.2	
Missing	2	1.5	9	2.4	<0.001
					
*Insignificant cancer* a					
Insignificant (Epstein)	47	39.2	28	8.2	<0.001
Insignificant (liberal)	73	56.2	70	19.7	<0.001
					
*Other pathological features*					
Vascular invasion	0	0.0	15	4.1	0.02
HGPIN	121	93.1	338	91.8	0.65
Seminal Vesicle invasion	1	0.8	29	7.9	0.003
Perineural invasion	11	8.5	109	29.8	<0.001
					
*Prostatectomy findings*					
Single tumour	17	13.1	94	25.4	
Multiple tumours	113	86.9	276	74.6	0.004
Unifocal disease	24	18.5	67	18.1	
Multifocal cancers	106	81.5	303	81.9	0.93
					
*Tumour volume*					
Mean (s.d.)	1.51 (3.2) ml		4.04 (9.3) ml		<0.001
					
*Prostate volume* b					
Mean (s.d.)	42.8 (17.5) ml		31.4 (13.3) ml		<0.001
					
*Prostatectomy weight*					
Mean (s.d.)	56.5 (21.4) g		46.2 (17.2) g		<0.001

Abbreviations: HGPIN=high-grade prostatic intraepithelial neoplasia; s.d.=standard deviation.

a Histological definition from the prostatectomy specimen.

b Determined by transrectal ultrasound (TRUS) measurement.

*t*-test, *P*-value.

**Table 4:** Previous reports of prostate cancer size and location

**Reference**	**Center**	**Collection**	**Selection**	***N***	**Country**	**Solitary or unilateral (%)**
Villers *et al* (1992)	Single	Retro	None	234	United States	50
Miller and Cygan (1994)	Single	Retro	None	151	United States	44
Djavan *et al* (1999)	Single	Retro	None	308	Austria	33
Wise *et al* (2002)	Single	Retro	None	486	United States	17
Noguchi *et al* (2003)	Single	Retro	T1c disease	222	United States	24
Ng *et al* (2004)	Single	Retro	None	364	United States	15
Eichelberger and Cheng (2004)	Single	Retro	None	312	United States	15
Horninger *et al* (2004)	Single	Retro	PSA <4.0 ng ml^−1^	80	Austria	35
Cheng *et al* (2005)	Single	Retro	Small volume (<0.5 ml)	62	United States	31
Torlakovic *et al* (2005)	Single	Retro	None	46	Croatia	35
Muezzinoglu *et al* (2006)	Multi	Retro	None	947	United States, Turkey and Japan	27
Scales *et al* (2007)	Multi	Retro	Low risk^a^	261	United States	35
Mouraviev *et al* (2007)	Single	Retro	None	1184	United States	19
Tareen *et al* (2009)	Single	Retro	None	1467	United States	21
Polascik *et al* (2009)	Single	Retro	Low/intermediate risk^b^	538	United States	23
Ward *et al* (2009)	Single	Retro	Unilateral disease on biopsy	180	United States	17
This series	Multi	Prospective	None	525	United Kingdom	33
						
Mean						28

Abbreviation: PSA=prostate-specific antigen.

a T1c or T2a, PSA⩽10 ng ml^−1^, Gleason ⩽6 and less than three positive cores.

b ⩽T2c, PSA⩽10 and Gleason ⩽7.
